# Unidimensional Two-Way Continuous-Variable Quantum Key Distribution Using Coherent States

**DOI:** 10.3390/e23030294

**Published:** 2021-02-27

**Authors:** Yiming Bian, Luyu Huang, Yichen Zhang

**Affiliations:** State Key Laboratory of Information Photonics and Optical Communications, School of Electronic Engineering, Beijing University of Posts and Telecommunications, Beijing 100876, China; bianyiming@bupt.edu.cn (Y.B.); hly@bupt.edu.cn (L.H.)

**Keywords:** continuous-variable quantum key distribution, two-way scheme, unidimensional modulation, general two-mode attacks, optimal attack strategy

## Abstract

We propose a unidimensional two-way continuous-variable quantum key distribution protocol with coherent states, where the sender modulates a single quadrature of the coherent states rather than both quadratures to simplify the structure of a two-way system. Security analysis is performed with a general attack strategy, known as two-mode attack, which helps to reduce limitations in the analysis. The performance of the protocol under all accessible two-mode attacks at fixed distance is illustrated. Further, two typical two-mode attack strategies are obtained from it, which are one-mode attack strategy and optimal two-mode attack strategy. Between them, the one-mode attack is the simplest form of the two-mode attack, while the optimal two-mode attack is the most complicated one. Simulations show that though the system is simplified, the performance of the two-way protocol with unidimensional modulation is still comparable to that of the counterpart with Gaussian modulation even against the optimal two-mode attack when Eve’s ability is maximized. Thus, the proposed protocol simplifies the two-way system while guaranteeing its performance to a certain extent. Especially in a practical system with short transmission distance and high excess noise, the protocol has a good application prospect.

## 1. Introduction

Quantum key distribution (QKD) [1] is an effective way to ensure communication security and resist the impact of quantum computing on an encryption system [2,3]. Based on the principles of quantum mechanics, a QKD protocol enables two trusted parties, usually called Alice and Bob, to distill secret keys with unconditional security in theory. Over the last three decades, QKD has developed rapidly and spawned two branches, discrete-variable QKD and continuous-variable QKD (CV-QKD) [4,5]. Compared with the discrete-variable QKD, CV-QKD has higher compatibility of standard telecom components [6], which reduces the cost, improves the integration and facilitates the large-scale application of QKD [7,8,9,10,11,12]. In recent years, CV-QKD has gathered momentum: the transmission distance of CV-QKD has been further extended in commercial fibers [10], low loss optical fibers in laboratory [12], and the theory of CV-QKD has also been widely improved [13,14,15,16,17,18].

To enhance the tolerable excess noise of the CV-QKD system, the two-way CV-QKD protocol was proposed [19]. In this protocol, Bob sends states to Alice through the forward channel. After which, Alice couples her own states with the received states and then sends them back to Bob through the backward channel. In this way, the information of the secret key is loaded onto the coupled quantum state, and transferred from Alice to Bob. In order to obtain the key information, an eavesdropper, Eve, must eavesdrop on both channels at the same time, which directly increases the eavesdropping difficulty. The structure is more beneficial to Alice and Bob when the channel is too noisy compared to the one-way protocol. Afterwards, an improved two-way CV-QKD protocol has also been proposed to make the two-way system easier to implement by replacing Alice’s displacement operation with a beam splitter [20], which promotes the practical application of two-way protocol, and thus, has been widely studied [21,22,23,24,25,26,27]. Although the performance of the two-way protocol is better than that of the one-way protocol, the complexity of the protocol is fairly high: It takes up two quantum channels and requires Gaussian modulation in both *x* and *p* quadratures at both Alice’s and Bob’s side. Therefore, it is necessary to build a simpler and more feasible two-way scheme.

Reducing the number of the modulators by waiving the symmetry of modulation is one of the strategies to simplify the protocol, namely, the unidimensional (UD) modulation [28]. It simplifies the system by abandoning one quadrature of the modulations, and shows a comparable performance to the conventional Gaussian modulated ones under low noise conditions [28]. Until now, the feasibility of using UD modulation has been demonstrated in the squeezed state protocol [29,30] and the measurement-device-independent protocol [31,32]. Simultaneously, the analysis of UD CV-QKD protocol in practical situations has been improved [33], and the methods to enhance the performance of UD protocol have also been proposed [34,35]. In addition, with single amplitude modulator, the UD protocol has been tested experimentally [36]. Considering that, on the one hand, the using of UD modulation is conducive to the simplification of two-way system. On the other hand, the high anti-noise performance of the two-way system contributes to the compensation of the sensitivity to noise caused by UD modulation. A simplified two-way CV-QKD with UD modulation is expected to have good performance.

In this paper, a UD two-way CV-QKD protocol is proposed. UD modulation is used at Alice’s side instead of the conventional Gaussian modulation, which enables the two-way system to work with a single quadrature modulation. In this way, the complexity of the protocol and the number of quantum random numbers required are reduced. In this protocol, Bob still uses conventional Gaussian modulation to reduce the complexity of security analysis, and to avoid dramatic degradation of the protocol performance caused by multiple use of unidimensional modulation. For security analysis of a two-way protocol, a general two-mode attack strategy is used: Eve is assumed to have the ability to exploit the correlation between the modes used to attack the forward channel and backward channel. To illustrate the system performance against the general two-mode attack, we derive the expressions of the secret key rate of the protocol against general two-mode attacks and perform simulations of the protocol against all accessible two-mode attacks at fixed distance. Furthermore, two typical two-mode attack strategies, namely, the one-mode attack strategy and the optimal two-mode attack strategy, are obtained from the general two-mode attack, which are the simplest and the most complex cases of the general two-mode attack strategy, respectively. For the one-mode attack, there is no correlation between the two attack modes, while for the optimal two-mode attack, Eve is able to find the optimal attack correlations and minimize the performance of the protocol. Under these two attack strategies, performances of the protocol within secure transmission distances are simulated and compared with that of symmetrical modulated counterparts.

## 2. The Schemes of Unidimensional Two-Way CV-QKD Protocol

In this part, we first introduce the difference between UD modulation and symmetrical Gaussian modulation with the prepare-and-measure (PM) scheme of the UD two-way CV-QKD protocol. Then the entanglement-based (EB) scheme is illustrated, and the reason of using this modulation strategy is explained.

**Figure 1:**
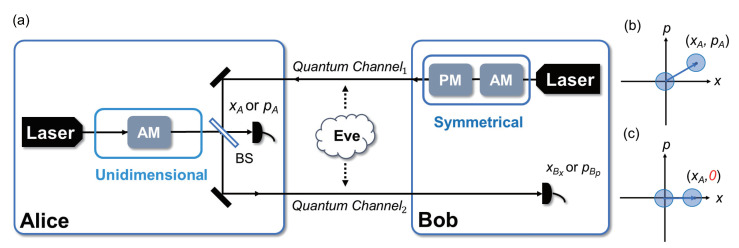
(Color online) (**a**) The prepare-and-measure (PM) scheme of the unidimensional (UD) two-way protocol. Here, the two quantum channels are fully controlled by Eve while she has no access to the apparatuses in Alice’s and Bob’s stations. (**b**) Symmetrical Gaussian modulated coherent state on a phase space. (**c**) UD modulated coherent state on a phase space, assuming only *x* quadrature was modulated, which means, the coherent state can only be shifted along *x* on the phase space. Here, $x_{A}$ and $p_{A}$ are two independent identically distributed Gaussian random variables, and the coordinates describe the centers of the shifted coherent states. (BS: beam splitter, AM: amplitude modulator, PM: phase modulator).

In terms of the structure of the protocol, as shown in Figure 1, the UD modulation is used at Alice’s side and the symmetrical modulation is used at Bob’s side. The most different part between the UD protocol and the symmetrical Gaussian modulated protocol is the number of the modulators. The symmetrical Gaussian modulation modulates *x* quadrature and *p* quadrature simultaneously, while the UD modulation modulates only one single quadrature (amplitude quadrature *x* or phase quadrature *p*). In this way, CV-QKD can be performed with only one modulator and the complexity of the protocol is reduced. Without loss of generality, the *x* quadrature is used in the discussion below.

**Figure 2:**
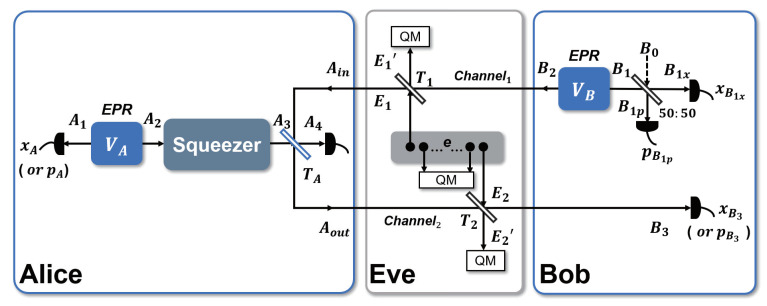
(Color online) The entanglement-based (EB) scheme of the UD two-way protocol against two-mode collective attacks, where Eve has full control of the quantum channels while she has no access to the apparatuses in Alice’s and Bob’s stations. At Alice’s side, one mode of the EPR pair is measured by homodyne detection while the other mode is sent to a squeezer, this part is equivalent to the PM scheme of UD modulation with coherent state. The blue beam splitter with a transmittance of $T_{A}$ is used to couple Alice’s state with Bob’s state. (QM: quantum memory, $B_{0}$ vacuum state).

The EB scheme of the UD two-way protocol is shown in Figure 2, and can be described as follows:

*Step 1.* Alice prepares an EPR state with variance $V_{A}$. She measures one mode $A_{1}$ with homodyne detection and sends the other mode $A_{2}$ to a squeezer, which is equivalent to the unidimensional modulation in the PM scheme [35]. At the same time, Bob prepares another EPR state with variance $V_{B}$. He keeps one mode $B_{1}$ and sends the other mode $B_{2}$ to Alice through $Channel_{1}$ where Eve may perform an attack.

*Step 2.* Alice couples the received mode $A_{in}$ with her own mode $A_{3}$ through a beam splitter, she then sends the coupling mode $A_{out}$ back to Bob through $Channel_{2}$ while measuring mode $A_{4}$ with homodyne detection for parameter estimation.

*Step 3.* Bob measures the retained mode $B_{1}$ with heterodyne detection to get $x_{{B_{1}}_{x}}$ and $p_{{B_{1}}_{p}}$, and the received mode $B_{3}$ is measured with homodyne detection by him to get $x_{B_{3}}$.

*Step 4.* Bob uses $x_{B_{x}}=x_{B_{3}}-k\;x_{{B_{1}}_{x}}$ to construct the estimator to Alice’s corresponding variable $x_{A}$. Here *k* is the parameter used to optimize Bob’s estimator of Alice’s corresponding value. Then the postprocessing including reconciliation and privacy amplification is performed.

It is essential to note that although the *p* quadrature is not modulated, it still needs to be measured sometimes to gather the properties of the channel in the *p* quadrature in order to ensure the security, and the impact of these measurements on secret key rate is considered negligible [28].

The cost of the simplification by using UD modulation is that the *p* quadrature is not modulated, which results in the impossibility of estimating the correlation and the channel transmittance in *p*. Thus, after passing the quantum channel, an unknown parameter $Cor_{p}$ is introduced into the security analysis to represent the unknown correlation in *p* quadrature [28], which has a negative impact on the performance of the protocol. In addition to the scheme shown in Figure 1, UD modulation can also be used solely at Bob’s side (while Alice still uses symmetrical Gaussian modulation) or both at Alice’s and Bob’s sides. However, by calculation, these two schemes introduce one or two more unknown parameters into the covariance matrix compared with the scheme shown in Figure 1, mainly because the two-way scheme amplifies the loss of correlation caused by UD modulation in these cases. Considering that more unknown parameters result in more loopholes open to eavesdropper and make numerical simulation in security analysis difficult, in the following security analysis, we focus on the scheme with UD modulation solely used at Alice’s side.

## 3. Security Analysis of the Protocol Against Two-Mode Attack

In the security analysis of two-way protocols, one-mode attack strategy is usually used, which assumes that there are no correlations between the two modes of Eve used for attack. However, it has some limitations: It is a special attack structure which can not describe the other attack strategies, and it may not be optimal for the UD two-way protocol. Thus, it is essential to use the general two-mode attack strategies to make a comprehensive analysis. In this section, we first introduce the two-mode attack strategy used by Eve. Then, the process of calculating the secret key rate of the protocol is deduced. To obtain the worst secret key rate and analyze the protocol in the strictest case, we use a double pessimistic worst-case assumption, which is composed of two parts: The first part is used to obtain the optimal attack strategy from the two-mode attack, and the second part is used to get the worst unknown correlation parameters in *p* quadrature.

### 3.1. Two-Mode Attack Strategy

The entanglement-based (EB) scheme of two-mode attack strategy is shown in Figure 2. For a two-way system, Eve needs to attack both quantum channels, which makes it more difficult for her to carry out eavesdropping but also enables her to maximize the information eavesdropped by optimizing the correlation of $E_{1}$ and $E_{2}$ [26,37]. If we simply use a one-mode attack for analysis, the correlation of the two attacking modes cannot be reflected, therefore, a general two-mode attack is used for the following analysis to evaluate the protocol performance comprehensively and to get the optimal attack strategy.

As shown in Figure 2, Eve mixes the modes $B_{2}$ and $A_{out}$ with $E_{1}$ and $E_{2}$ in $Channel_{1}$ and $Channel_{2}$, through beam splitters with transmissivity $T_{1}$ and $T_{2}$. The other modes *e*, together with $E_{1}$ and $E_{2}$, form a whole, and can be globally described by a pure Gaussian state. Afterwards, $E_{1}^{\prime }$, $E_{2}^{\prime }$ and *e* are stored in a quantum memory and measured at the end of the protocol. The covariance matrix of $E_{1}$ and $E_{2}$ has the normal form

(1) $$\gamma_{E_{1}E_{2}}=\left(\begin{array}{cc} V_{E_{1}}\cdot I_{2} & C_{E_{1}E_{2}}\\ C_{E_{1}E_{2}} & V_{E_{2}}\cdot I_{2} \end{array}\right)$$

where $C_{E_{1}E_{2}}=\left(\begin{array}{cc} C_{x} & 0\\ 0 & C_{p} \end{array}\right)\;$ is the correlation parameter matrix between $E_{1}$ and $E_{2}$, $I_{2}=\left(\begin{array}{cc} 1 & 0\\ 0 & 1 \end{array}\right).$ $V_{E_{1}}=1+\frac{T_{1}\epsilon }{1-T_{1}}$, $V_{E_{2}}=1+\frac{T_{2}\epsilon }{1-T_{2}}$ are the variances of the thermal noise. Here $T_{1}$ and $T_{2}$ are given by $T_{1}=T_{2}=10^{-\alpha L/10}$, and α=0.2dB/km is the loss coefficient of the optical fibers. ϵ is the excess noise, for simplicity, we assume that the excess noise of the two channels are the same. Thus, when ϵ and *L* are fixed, $V_{E_{1}}$ and $V_{E_{2}}$ are also determined accordingly. At this point, Eve’s attack strength is totally determined by $C_{x}$ and $C_{p}$.

To explore the performance of the protocol in the worst case, the ability of Eve is maximized: We assume that Eve is able to determine $C_{x}$ and $C_{p}$ arbitrarily within the limits of physical existence condition [26,37,38], and she can further find the optimal attack parameters $C_{E_{1}E_{2}}^{worst}$ to minimize the performance of the protocol. This is the first part of our double pessimistic worst-case assumption.

The bound of $C_{x}$ and $C_{p}$ can be written as

(2) $$\gamma_{E_{1}E_{2}}>0,\;\;v_{-}\geq 1,$$

where $v_{-}$ is the least symplectic eigenvalue of $\gamma_{E_{1}E_{2}}$, written as

(3) $$\begin{array}{cc} \; & v_{-}=\sqrt{0.5(\Delta -\sqrt{\Delta^{2}-4\det\left(\gamma_{E_{1}E_{2}}\right)})}, \end{array}$$

with

(4) $$\begin{array}{c} \Delta =V_{E_{1}}^{2}+V_{E_{2}}^{2}+2C_{x}C_{p}. \end{array}$$

From $\gamma_{E_{1}E_{2}}>0$, the following restriction is derived,

(5) $$|C_{x}|\;<\sqrt{V_{E_{1}}V_{E_{2}}},\;\;|C_{p}|\;<\sqrt{V_{E_{1}}V_{E_{2}}}.$$

### 3.2. The Secret Key Rate of the Protocol

In reverse reconciliation, the secret key rate *K* of the protocol against two-mode Gaussian attacks is given by [39]

(6) K=βI(A:B)−χ(B:E),

where β is the reconciliation efficiency, I(A:B) is the classical mutual information between Alice and Bob, and χ(B:E) is the upper bound of the von Neumann entropy between Eve and Bob, called the Holevo bound [40]. I(A:B) and χ(B:E) can be derived from the covariance matrix $\gamma_{A_{1}A_{4}B_{1}B_{3}}$ and $\gamma_{A_{1}A_{4}B_{6}B_{1p}}^{B_{x}}$, which describes the system after the modes go through the channels, and the system after the measurements, respectively. Here, $I_{AB}$ is given by

(7) $$I\left(A:B\right)=0.5\;log_{2}\left(V_{A_{1}}/V_{A1}^{x_{B_{x}}}\right),$$

where $V_{A_{1}}=\gamma_{A_{1}A_{4}B_{6}B_{1p}}^{B_{x}}\left(2,\;2\right)$ and $V_{A1}^{x_{B_{x}}}=\gamma_{A_{1}A_{4}B_{6}B_{1p}}^{B_{x}}\left(1,\;1\right)$. Assuming that Eve can purify Alice and Bob’s system, χ(B:E) is given by

(8) $$\begin{array}{c} \chi \left(B:E\right)=S\left(\rho_{A_{1}A_{4}B_{1}B_{3}}\right)-S\left(\rho_{A_{1}A_{4}B_{6}B_{1p}}^{x_{B_{x}}}\right)=\sum_{i=1}^{4}G\left(\frac{\lambda_{i}-1}{2}\right)-\sum_{j=5}^{8}G\left(\frac{\lambda_{j}-1}{2}\right), \end{array}$$

where $\lambda_{1∼4}$ and $\lambda_{5∼8}$ are the symplectic eigenvalues of the covariance matrix $\gamma_{A_{1}A_{4}B_{1}B_{3}}$, and $\gamma_{A_{1}A_{4}B_{6}B_{1p}}^{x_{B_{x}}}$.

The derivation of the above two matrices is as follows. First, the covariance matrix which describes the states $A_{1}$, $A_{2}$, $B_{1}$ and $B_{2}$ is given by

(9) $$\begin{array}{c} \gamma_{A_{1}A_{2}B_{1}B_{2}}=\left(\begin{array}{cccc} V_{A}\cdot I_{2} & \sqrt{V_{A}^{2}-1}\cdot \sigma_{z} & 0\cdot I_{2} & 0\cdot I_{2}\\ \sqrt{V_{A}^{2}-1}\cdot \sigma_{z} & V_{A}\cdot I_{2} & 0\cdot I_{2} & 0\cdot I_{2}\\ 0\cdot I_{2} & 0\cdot I_{2} & V_{B}\cdot I_{2} & \sqrt{V_{B}^{2}-1}\cdot \sigma_{z}\\ 0\cdot I_{2} & 0\cdot I_{2} & \sqrt{V_{B}^{2}-1}\cdot \sigma_{z} & V_{B}\cdot I_{2} \end{array}\right), \end{array}$$

where $\sigma_{z}=\left(\begin{array}{cc} 1 & 0\\ 0 & -1 \end{array}\right),$ $V_{A}$ and $V_{B}$ are the variances of Alice’s EPR state and Bob’s EPR state. Then, after $A_{2}$ goes through the squeezer, which is equivalent to the UD modulation in PM scheme, the covariance matrix which describes the state $A_{1}$, $A_{3}$ is given by

(10) $$\begin{array}{c} \gamma_{A_{1}A_{3}}=\left(\begin{array}{cccc} V_{A} & 0 & \sqrt{V_{A}\left(V_{A}^{2}-1\right)} & 0\\ 0 & V_{A} & 0 & -\sqrt{\frac{\left(V_{A}^{2}-1\right)}{V_{A}}}\\ \sqrt{V_{A}\left(V_{A}^{2}-1\right)} & 0 & V_{A}^{2} & 0\\ 0 & -\sqrt{\frac{\left(V_{A}^{2}-1\right)}{V_{A}}} & 0 & 1 \end{array}\right). \end{array}$$

After transmission through quantum channels, following the channel transmission relationships as below

(11) $$\begin{array}{c} \left\{\begin{array}{cc} A_{in}=\sqrt{T_{1}}B_{2}+\sqrt{1-T_{1}}E_{1}, & \\ A_{4}=\sqrt{T_{A}}A_{3}-\sqrt{1-T_{A}}A_{in}, & \\ A_{out}=\sqrt{T_{A}}A_{in}+\sqrt{1-T_{A}}A_{3}, & \\ B_{3}=\sqrt{T_{2}}A_{out}+\sqrt{1-T_{2}}E_{2}. \end{array}\right. \end{array}$$

The covariance matrix which describes the states $A_{1}$, $A_{4}$, $B_{1}$ and $B_{3}$ is given by

(12) $$\gamma_{A_{1}A_{4}B_{1}B_{3}}=\left(\begin{array}{cccc} V_{A}\cdot I_{2} & C_{A_{1}A_{4}} & 0\cdot I_{2} & C_{A_{1}B_{3}}\\ C_{A_{1}A_{4}} & \gamma_{A_{4}} & C_{A_{4}B_{1}} & C_{A_{4}B_{3}}\\ 0\cdot I_{2} & C_{A_{4}B_{1}} & V_{B}\cdot I_{2} & C_{B_{1}B_{3}}\\ C_{A_{1}B_{3}} & C_{A_{4}B_{3}} & C_{B_{1}B_{3}} & \gamma_{B_{3}} \end{array}\right),$$

where

(13) $$\begin{array}{c} \gamma_{A_{4}}=\left(\begin{array}{cc} T_{A}\;{V_{A}}^{2}+\left[V_{E}\;\left(T_{1}-1\right)-T_{1}\;V_{B}\right]\;\left(T_{A}-1\right) & 0\\ 0 & T_{A}+\left[V_{E}\;\left(T_{1}-1\right)-T_{1}\;V_{B}\right]\;\left(T_{A}-1\right) \end{array}\right), \end{array}$$

and

(14) $$\begin{array}{c} \gamma_{B_{3}}=\left(\begin{array}{cc} V_{{B_{3}}_{x}} & 0\\ 0 & V_{{B_{3}}_{p}} \end{array}\right). \end{array}$$

Here, $V_{{B_{3}}_{x}}$ and $V_{{B_{3}}_{p}}$ and the variances of the *x* quadrature and *p* quadrature of $B_{3}$, and have the following form:

(15) $$\begin{array}{cc} V_{{B_{3}}_{x}}= & 2\;C_{x}\;\sqrt{T_{2}\;T_{A}\;\left(T_{1}-1\right)\;\left(T_{2}-1\right)}-V_{E}\;\left(T_{2}-1\right)\\ & -T_{2}\;{V_{A}}^{2}\;\left(T_{A}-1\right)-T_{2}\;T_{A}\;V_{E}\;\left(T_{1}-1\right)+T_{1}\;T_{2}\;T_{A}\;V_{B}, \end{array}$$

and

(16) $$\begin{array}{cc} V_{{B_{3}}_{p}}= & 2\;C_{p}\;\sqrt{T_{2}\;T_{A}\;\left(T_{1}-1\right)\;\left(T_{2}-1\right)}-V_{E}\;\left(T_{2}-1\right)\\ \; & -T_{2}\;\left(T_{A}-1\right)-T_{2}\;T_{A}\;V_{E}\;\left(T_{1}-1\right)+T_{1}\;T_{2}\;T_{A}\;V_{B}. \end{array}$$

The correlation between modes $A_{1}$ and $A_{4}$ is represented by

(17) $$\begin{array}{c} C_{A_{1}A_{4}}=\sqrt{T_{A}\left({V_{A}}^{2}-1\right)}\;\left(\begin{array}{cc} \sqrt{V_{A}} & 0\\ 0 & -\sqrt{1/V_{A}} \end{array}\right), \end{array}$$

and the correlation between modes $A_{1}$ and $B_{3}$ is represented by

(18) $$\begin{array}{c} C_{A_{1}B_{3}}=\left(\begin{array}{cc} \sqrt{V_{A}\left(\;{V_{A}}^{2}-1\;\right)T_{2}\;\left(1-T_{A}\right)} & 0\\ 0 & Cor_{p} \end{array}\right). \end{array}$$

Here, as discussed in Section 2, $Cor_{p}$ is used to represent the unknown correlation in *p* quadrature and assumed to be bounded by the physical existence condition, which is given by the Heisenberg uncertainty principle. In the strictest case, to get the worst protocol performance degradation caused by the loss of correlation parameters, the worst correlation parameter $Cor_{p}^{worst}$ which minimize the secret key rate is used for security analysis [28,29,30,31,32,33,34,35,36]. In this way, we get the worst key rate under UD modulation, and this is the second part of our double pessimistic worst-case assumption in terms of unknown correlation parameters.

The correlations between modes $A_{4}$ and $B_{1}$, and between $A_{4}$ and $B_{3}$, are represented by $C_{A_{4}B_{1}}$ and $C_{A_{4}B_{3}}$, written as below:

(19) $$\begin{array}{c} C_{A_{4}B_{1}}=\sqrt{\left({V_{B}}^{2}-1\right)\;\left(1-T_{A}\right)\;T_{1}}\left(\begin{array}{cc} -1 & 0\\ 0 & 1 \end{array}\right), \end{array}$$

(20) $$\begin{array}{c} C_{A_{4}B_{3}}=\left(\begin{array}{cc} C_{A_{4}{B_{3}}_{x}} & 0\\ 0 & C_{A_{4}{B_{3}}_{p}} \end{array}\right), \end{array}$$

with

(21) $$\begin{array}{c} C_{A_{4}{B_{3}}_{x}}=\sqrt{T_{2}T_{A}\left(1-T_{A}\right)}\left[{V_{A}}^{2}-V_{B}\;T_{1}-V_{E}\;\left(1-T_{1}\right)\right.\left.-C_{x}\sqrt{\left(1-T_{2}\right)\;\left(1-T_{1}\right)/\left(T_{2}T_{A}\right)}\;\right], \end{array}$$

and

(22) $$\begin{array}{c} C_{A_{4}{B_{3}}_{p}}=\sqrt{T_{2}T_{A}\left(1-T_{A}\right)}\left[1-V_{B}\;T_{1}-V_{E}\;\left(1-T_{1}\right)\right.\left.-C_{p}\sqrt{\left(1-T_{2}\right)\left(1-T_{1}\right)/\left(T_{2}T_{A}\right)}\;\right]. \end{array}$$

$C_{B_{1}B_{3}}$ represents the correlation between modes $B_{1}$ and $B_{3}$, and can be written as

(23) $$\begin{array}{c} C_{B_{1}B_{3}}=\sqrt{\left(\;{V_{B}}^{2}-1\;\right)T_{1}\;T_{2}\;T_{A}}\left(\begin{array}{cc} 1 & 0\\ 0 & -1 \end{array}\right). \end{array}$$

In the formulas above, $T_{A}$ is the transmission efficiency of the beam splitter at Alice’s side. With heterodyne detection of $B_{1}$ at Bob’s side, we can get $\gamma_{A_{1}A_{4}B_{3}B_{1x}B_{1p}}$, which is given by

(24) $$\begin{array}{c} \gamma_{A_{1}A_{4}B_{3}B_{1x}B_{1p}}=\gamma_{BS}\cdot \left(\gamma_{A_{1}A_{4}B_{3}B_{1}}⊕I_{2}\right)\cdot \gamma_{BS}^{T}\;, \end{array}$$

where $\gamma_{BS}=\left(I_{6}⊕\gamma_{BS}^{0}\right)$, $I_{6}$ is an identity matrix of 6×6, and $\gamma_{BS}^{0}=\sqrt{\eta }\left(\begin{array}{cc} I_{2} & I_{2}\\ -I_{2} & I_{2} \end{array}\right).$ For heterodyne detection, η=0.5. In the same way as the original two-way system for covariance matrix derivation [20], the construction of the estimator mentioned in Section 2 can be simplified with a Control-Not gate, thus $\gamma_{A_{1}A_{4}B_{1p}B_{6}B_{x}}$ is given by

(25) $$\gamma_{A_{1}A_{4}B_{1p}B_{6}B_{x}}=\Gamma^{T}\cdot \gamma_{A_{1}A_{4}B_{1p}B_{1x}B_{3}}\cdot \Gamma ,$$

where

(26) $$\Gamma =I_{6}⊕\Gamma_{x},$$

and

(27) $$\Gamma_{x}=\left(\begin{array}{cccc} 1 & 0 & -k & 0\\ 0 & 1 & 0 & 0\\ 0 & 0 & 1 & 0\\ 0 & k & 0 & 1 \end{array}\right)$$

represents a Control-Not gate, $k=\sqrt{2T_{A}T_{1}T_{2}\frac{V_{B}-1}{V_{B}+1}}$ is used to optimize the performance of system.

Finally we get $\gamma_{A_{1}A_{4}B_{6}B_{1p}}^{x_{B_{x}}}$, which is given by

(28) $$\gamma_{A_{1}A_{4}B_{6}B_{1p}}^{x_{B_{x}}}=\gamma_{A_{1}A_{4}B_{6}B_{1p}}-\sigma_{A_{1}A_{4}B_{6}B_{1p}B_{x}}^{T}\cdot H_{hom}\cdot \sigma_{A_{1}A_{4}B_{6}B_{1p}B_{x}},$$

where $H_{hom}={\left(X\cdot \gamma_{B_{x}}\cdot X\right)}^{MP}$, $X=\left(\begin{array}{cc} 1 & 0\\ 0 & 0 \end{array}\right)$ and MP means the Moore–Penrose pseudo-inverse of a matrix. The matrices $\gamma_{A_{1}A_{4}B_{6}B_{1p}}$, $\sigma_{A_{1}A_{4}B_{6}B_{1p}B_{x}}$, $\gamma_{B_{x}}$ are derived from $\gamma_{A_{1}A_{4}B_{6}B_{1p}B_{x}}$ in the following way:

(29) $$\begin{array}{c} \gamma_{A_{1}A_{4}B_{6}B_{1p}B_{x}}=\left(\begin{array}{cc} \gamma_{A_{1}A_{4}B_{6}B_{1p}} & \sigma_{A_{1}A_{4}B_{6}B_{1p}B_{x}}^{T}\\ \sigma_{A_{1}A_{4}B_{6}B_{1p}B_{x}} & \gamma_{B_{x}} \end{array}\right). \end{array}$$

So far, we get the two matrices needed to calculate the secret key rate, and here the secret key rate can be written as a function form as below:

(30) $$K=K(\beta ,\epsilon ,V_{A},V_{B},Cor_{p},C_{x},C_{p},T_{A},L).$$

From Equation (30), the parameters that determines the secret key rate are shown. Compared with the analysis of the symmetrical modulated two-way protocols [20], three more parameters are introduced, which are $Cor_{p}$, $C_{x}$ and $C_{p}$, respectively. Here, different values of $Cor_{p}$ represent different correlations of $A_{1}$ and $B_{3}$ in *p* quadrature, and $C_{x}$ and $C_{p}$ represent different two-mode attack strategies. Furthermore, the worst-case secret key rate $K^{worst}$ can be obtained by using $Cor_{p}^{worst}$ and $C_{E_{1}E_{2}}^{worst}$ instead of $Cor_{p}$ and $\left(C_{x},C_{p}\right)$, where $Cor_{p}^{worst}$ and $C_{E_{1}E_{2}}^{worst}$ can be obtained by simulations. At this point, the theoretical derivation of security analysis is finished.

## 4. Simulation and Discussion

In this section, we first show the performance of the UD two-way protocol against all accessible two-mode attacks at fixed distance, which is the basis of the analysis. Then, from the general two-mode attacks, two typical two-mode attack strategies are obtained, which are one-mode attack strategy and the optimal two-mode attack strategy. The simulations of the protocol against these two attack strategies are performed, which is conducive to the accurate analysis of the protocol performance. For all the simulations, $Cor_{p}^{worst}$ is used, which can be obtained from the comparison of $K(Cor_{p})$, when all the parameters except for $Cor_{p}$ are fixed and $Cor_{p}$ traverses within the physical existence constraint. This is a reflection of the pessimistic worst-case assumption of the unknown correlation parameters in the simulations. Ideal cases and practical cases are mainly simulated, with reconciliation efficiency of 1 and 0.956 [41,42], and variance of 1000 and 5, respectively.

**Figure 3:**
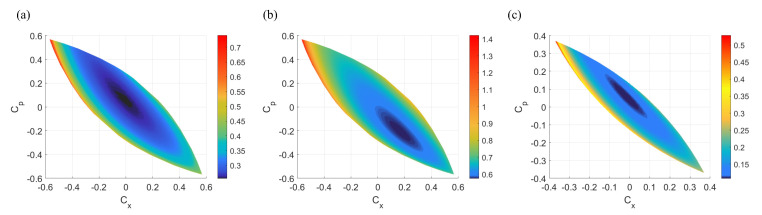
(Color online) Secret key rate of UD two-way CV-quantum key distribution (QKD) protocol under all accessible two-mode attacks under different situations. (**a**) At 5 km while V=5, β=0.956, ϵ=0.04. (**b**) At 5 km while $V=10^{3}$, β=1, ϵ=0.04. (**c**) At 10 km while V=5, β=0.956, ϵ=0.04. (For all simulations $T_{A}=0.8$).

As shown in Figure 3, on a $C_{x}$, $C_{p}$ plane, each point represents a two-mode attack strategy of Eve, the region that resembles an olive pit represents the physical existing two-mode attack, which means that Eve can choose these pairs of $C_{x}$ and $C_{p}$ for two-mode attack at the fixed parameters of the protocol. When *L* and ϵ are fixed, as shown in Figure 3a,b, the boundaries of the regions are same, but the distribution of the secret key rate varies with modulation variance and reconciliation efficiency. As the distance increases, as shown in Figure 3a,c, the region will gradually shrink, which means the two-mode attack is limited. What is more, the point (0,0) on the $C_{x}$, $C_{p}$ plane represents the one-mode attack, which is the simplest case of two-mode attacks.

Despite the different secret key rate distributions, these two-mode attack simulations still have one thing in common, that is, all of them have a minimum secret key rate center, shown in dark blue in the figure. This confirms that Eve can achieve a set of optimal two-mode attack parameters $C_{E_{1}E_{2}}^{worst}$ to get the most information, which minimizes the performance of the protocol and can be obtained from the comparison of $K(C_{x},C_{p})$, when all the parameters except for $\left(C_{x},C_{p}\right)$ are fixed and $\left(C_{x},C_{p}\right)$ traverses within the physical existence constraint. Thus, it requires us to perform two-mode attack at each distance and obtain the optimal attack parameters in security analysis to avoid overestimating the performance of the protocol. In this way, the performance of the protocol under the optimal two-mode attack is obtained and the worst performance of the UD two-way CV-QKD protocol is reflected, which is a representation of the pessimistic worst-case assumption of two-mode attack in the simulations.

**Figure 4:**
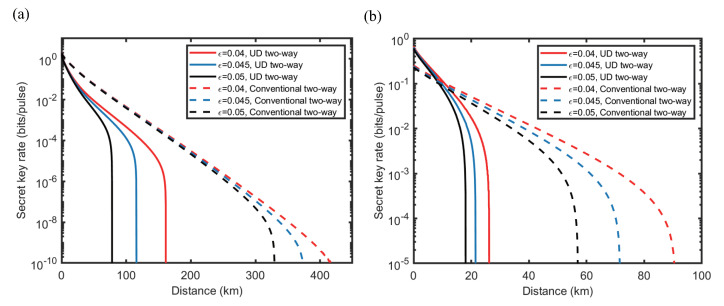
(Color online) Comparisons of secret key rates between UD two-way CV-QKD protocol (solid line) and a sub-protocol ($Het-Hom_{M}$) of the conventional symmetrical Gaussian modulated two-way CV-QKD protocol family [20] (dashed line), under ideal and practical situations. The attack strategies against both protocols are one-mode attack. (**a**) Ideal situations where $V=10^{3}$, β=1, ϵ=0.04 (red line), 0.045 (blue line) and 0.05 (black line). (**b**) Practical situations where V=5, β=0.956, ϵ=0.04 (red line), 0.045 (blue line), 0.05 (black line). (For both protocols $T_{A}=0.8$).

**Figure 5:**
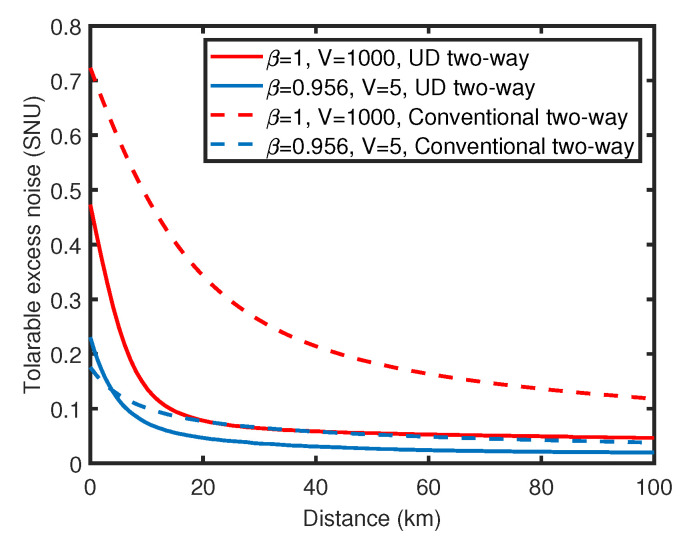
(Color online) Comparison of tolerable excess noise between UD two-way CV-QKD protocol (solid lines) and the sub-protocol ($Het-Hom_{M}$) of the conventional symmetrical Gaussian modulated two-way protocol family (dashed lines), the red lines represent the ideal situations and the blue lines represent the practical situations. (The attack strategies against both protocols are one-mode attack, and for both protocols $T_{A}=0.8$).

Against one-mode attack, the secret key rate and tolerable excess noise of UD two-way CV-QKD protocol is simulated and compared with the conventional symmetrical Gaussian modulated two-way CV-QKD protocol ($Het-Hom_{M}$) [20] in ideal case and practical case, as shown in Figure 4 and Figure 5. Here, $Het-Hom_{M}$ represents a two-way protocol with coherent state and homodyne detection at Bob’s side. Compared with the conventional two-way protocol, the performance of the UD two-way protocol is reduced to some extent because of the missing of information in *p* quadrature, which is similar to the performance degradation caused by UD modulation in one-way scheme [28]. In terms of the secret key rate, in the ideal case shown in Figure 4a, the key rate attenuation of the UD two-way protocol is acceptable when the excess noise is low. When β=1 and ϵ=0.04, in the range of 50 km to 150 km, the key rate of UD two-way protocol is approximately 1 to 1.5 orders of magnitude lower than the original protocol. In the practical case shown in Figure 4b, the performance gap is smaller, in the range of 0 km to 10 km, the secret key rate of UD two-way protocol is even higher than that of the original two-way protocol. Here, the outperformance of the UD two-way protocol in the practical case at short distances is mainly due to the fact that the modulation variance *V* is not optimal for the two protocols under the practical simulation parameters. The optimal modulation variance of the UD two-way protocol is about 10, while it is more than 20 for the original ones. This property limits the performance of the original two-way protocol when the modulation variance is low, and thus, causes the outperformance of the UD two-way protocol. In the aspect of the tolerable excess noise, as shown in Figure 5, the performance of the UD two-way protocol is similar to that of the original two-way protocol in practical, and is acceptable in the ideal conditions especially when the transmission distance is long. At 80 km, in the ideal case, the tolerable excess noise of UD two-way protocol is less than 0.1 lower compared with the original two-way protocol, and the performance gap is further reduced with the increase of distance.

**Figure 6:**
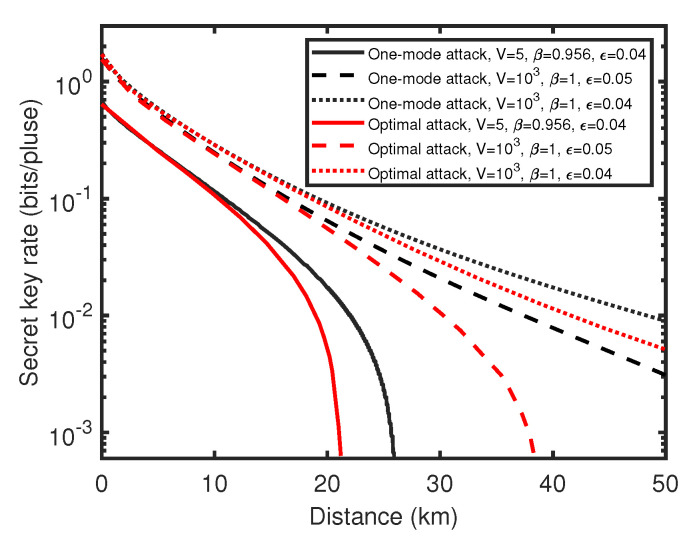
(Color online) Comparison of secret key rate between UD two-way CV-QKD protocol against the optimal two-mode attack (red line) and the one-mode attack (black line). Here, the solid line represents the practical situation, the dashed line and the dotted line represent the ideal situation. (For both cases $T_{A}=0.8$).

By using the optimal two-mode attack parameters $C_{E_{1}E_{2}}^{worst}$ and the worst correlation parameters $Cor_{p}^{worst}$, we get the secret key rate simulation of the UD two-way CV-QKD protocol against the optimal two-mode attack, which is shown in Figure 6. Against the optimal two-mode attack, the performance of the UD two-way CV-QKD protocol is further reduced, however, compared with the performance of the protocol against one-mode attack, the performance loss is not serious when the excess noise is low. Especially under the practical case where ϵ=0.04, the loss of transmission distance is less than 5 km. This means, though Eve is able to utilize the two-mode correlation between $E_{1}$ and $E_{2}$ to optimize the attack strategy, the enhancement of the attack strength is limited. These show that the UD two-way protocol has good resistance to two-mode attacks under low noise condition, and its lower performance limit is acceptable. Thus, the lower bound of the UD two-way CV-QKD protocol is obtained.

## 5. Conclusions

In this paper, a two-way CV-QKD protocol with UD modulation is proposed, and we focus on the case where UD modulation and symmetrical modulation are used at the sender’s (Alice’s) side and receiver’s (Bob’s) side, respectively. The expressions of the secret key rate of the protocol against general two-mode attacks are derived, and the simulations of the protocol against all accessible two-mode attacks are performed. Here, we use a double pessimistic worst-case assumption to analyze the protocol in the strictest case. Through this assumption, the missing correlation parameters and the two-mode attack strength are limited to the worst case, which minimize the protocol performance. Furthermore, one-mode attack strategy and optimal two-mode attack strategy are obtained from the general two-mode attacks, and the lower bound of the protocol performance is shown.

Through simulations, although the performance of UD two-way protocol is partially reduced, which is the price of the protocol simplification, it is still acceptable compared to that of the symmetrical Gaussian modulated counterpart. In practical cases, the performance of the UD two-way CV-QKD protocol is similar to the symmetrical Gaussian modulated counterpart, and at a short transmission distance, the UD two-way protocol even performs better. Therefore, it is reasonable to use unidimensional modulation in a two-way scheme to simplify the system, which expands the application scenario of the two-way CV-QKD system. The future research on the protocol will focus on the other UD modulation strategy of the two-way scheme, such as using UD modulation singly at Bob’s side or at both Alice’s and Bob’s side. Multiple pessimistic worst-case assumptions can be used for security analysis of those cases, and the study of the analytic solution of the security bound is also helpful to simplify the security analysis of unidimensional two-way CV-QKD protocols. 

## Data Availability

Data sharing not applicable.
